# Construction of Atomically Thin Boron Films on Si Heterojunctions Using a First Principles Approach

**DOI:** 10.3390/ma19050952

**Published:** 2026-02-28

**Authors:** Piet Xiaowen Fang, Stoyan Nihtianov, Changming Fang

**Affiliations:** 1 Electronic Instrumentation Lab, Faculty of Electrical Engineering, Mathematics and Computer Science, TU Delft, Mekelweg 4, 2628 CD Delft, The Netherlands; lele.fang@gmail.com (P.X.F.);; 2 Brunel Centre for Advanced Solidification Technology (BCAST), Brunel University London, Uxbridge UB8 3PH, UK

**Keywords:** ab initio molecular dynamics simulation, ultraviolet photodiode, amorphous boron thin layer, PureB devices, a-B/Si interface chemistry

## Abstract

Deposition of amorphous boron (a-B) onto Si substrates via chemical decomposition of B_2_H_6_ molecules produces a-B/Si, heterojunctions which are the core parts of photodetectors used in vacuum ultraviolet (VUV) and potentially in extreme ultraviolet (EUV) lithography. However, fundamental questions regarding the limit on the thickness of the deposited a-B thin films and the intrinsic electronic nature of the B atoms adjacent to the Si substrate remain unanswered. Here we investigated the local structural and electronic properties of atomic-thin a-B layers at the Si{001} substrates using ab initio molecular dynamics (AIMD) techniques. The investigation revealed a rich variety of local chemical bonding and consequently interfacial electronic properties. For thin a-B layer(s)/Si systems, most of the a-B atoms at the interface formed (-B-Si-B-Si-) chains on the Si{001} surface. These B atoms were found to occupy the positions of the missing Si atoms and were bonded to the surficial Si atoms. The surficial Si atoms predominantly have two B neighbors. Localized defect states at the Fermi level for the interfacial Si and B atoms were found in the pseudo-gap. These states have a major influence on the electrical properties of the device. The predicted minimum thickness of the a-B films is about 1 to 2 nm, a useful metric for the manufacturing of a-B/Si devices. The information obtained here further helps us to understand the working mechanisms of a-B/Si interfaces for photon detection and constructing new core devices for potential applications in the field of metal/semiconductor heterojunctions for photon detection, photovoltaics, Schottky diodes and semiconductor devices.

## 1. Introduction

In recent years, the semiconductor industry has steadily been moving on from 193 nm based deep ultraviolet (DUV) lithography technology towards the EUV (extreme ultraviolet, 13.5 nm wavelength) spectrum to reach smaller node sizes. However, the development of a new generation of photolithography devices capable of detecting photons at these wavelengths is dependent on the ability to detect EUV photons. The amorphous boron (a-B) on silicon (a-B/Si) photodiode has been known to be a good detector for both DUV and EUV photons due to the shallow depletion region and the nanometer-thin—or even atomic-thin—capping boron layer. Experiments have revealed that these devices are able to reach a quantum efficiency of approximately 46% when used to detect 193 nm wavelength photons, while at 13.5 nm the device approaches the theoretical limit [1,2,3,4]. The a-B/Si device is furthermore shown to be stable under prolonged exposure to UV photons and low-energy electrons, as well as environmental factors such as temperature [4]. As a result, the a-B/Si photodiodes compare very favorably to commercially available Si-based VUV and EUV [2,4] and are already used in nanolithography devices. In the interest of further development, studying the fundamental properties, e.g., the limit of the thickness of the deposited a-B layer and local structural and electronic properties, is necessary to reach the level of comprehensive understanding needed to obtain devices with more desirable properties.

Experimental efforts on a-B/Si photodiodes have been primarily focused on the creation process, through experimentation with the deposition temperatures [3,5], thickness [5] and other conditions such as oxidation at the interfaces [6], as well as electric and optoelectronic properties measurements [4]. Recently, Hassan et al. annealed boron thin films to fabricate low-defect p^+^ Si regions [7]. Moreover, High-Resolution Transmission Electron Microscopy (HR-TEM) measurements provided clarity on the local structure of the a-B/Si heterojunctions—specifically, the observed sharp interface that arises at low deposition temperatures (~400 °C)—which did not fit a δ-doping model [8,9], as this model relies on diffusion of B atoms, which requires higher temperatures due to the relatively high kinetic barriers [8]. These findings indicate a need to build a new model to explain the electronic properties of this heterojunction. However, the obtained information about local structures and B distribution via the HR-TEM images is ‘averaged’ over the atoms across the samples. Information on the local structures, chemical interactions and limits of a-B thickness on the electronic properties of the a-B/Si interfaces at an atomic level is necessary to understand the interface in a better and more comprehensive way and the working mechanisms behind the observed phenomena [4,8,9].

Theoretical approaches, especially parameter-free first-principles methods, are very useful to describe such systems at the required scale. Theoretical efforts have been made to study the chemical processes involved in the chemical decomposition of the B_2_H_6_ precursor, which has up until now yielded constructive information on the formation of B layers on Si at an atomic scale, in addition to its electrical properties [10,11]. It was found that the deposited B atoms and BH_n_ (n = 1 to 3) radicals introduce localized surface states which affect the electronic properties of these interfaces. Recent first-principles modeling has provided detailed information about local structures and interfacial interactions, as well as electronic properties of an a-B bulk on Si heterojunction [12]. Of particular interest is the local structure and ordering of the B atoms on top of the Si surface, as it was shown that certain coordination patterns (1 and 2 B atoms per Si atom for the {111} and {001} surfaces, respectively) are dominant at the interface. This observation has led to questions about the conditions under which these local chemical bonds are found, particularly whether this is a consequence of the thickness of the amorphous boron layer and how this influences the electronic properties of the interfaces, in addition to the intrinsic electronic nature of the a-B atoms adjacent to the Si substrates. Here we systematically analyze the effects of the thickness of amorphous B (from one to four atomic layers, from about 2 Å to 8 Å) on the local structural and electronic properties of the a-B/Si{001} systems using an ab initio molecular dynamics approach. We show the growth and structure of the heterojunction and the local ordering of the B atoms near the interface. We further discuss the effects of low B atom population on the condensation of an interfacial layer and compare the electronic properties of this interface to the bulk growth process of a-B. The knowledge obtained here regarding the growth mechanism of bulk a-B and the electronic properties is further applicable to the metal/semiconductor heterojunctions in general for various applications including photon detectors, photovoltaics, Schottky diodes and related semiconductor devices [4,13,14,15,16,17,18,19].

## 2. Simulation Methods

### 2.1. The a-B/Si{001} Interface Systems

To study the properties of an ideal a-B/c-Si system, we simulated the interfaces at the atomic scale using AIMD simulation techniques. We first individually built the components of the interfaces, which consist of the crystalline Si{001} substrates and the amorphous boron slabs of various atomic thicknesses. We started from a bulk a-B/Si{001} interface system, which has a tetragonal cell with an in-plane axis *a* = 3*a*_0_ (where *a*_0_ is the lattice parameter of the Si cell with consideration of thermal expansion) [20,21].

**Figure 1:**
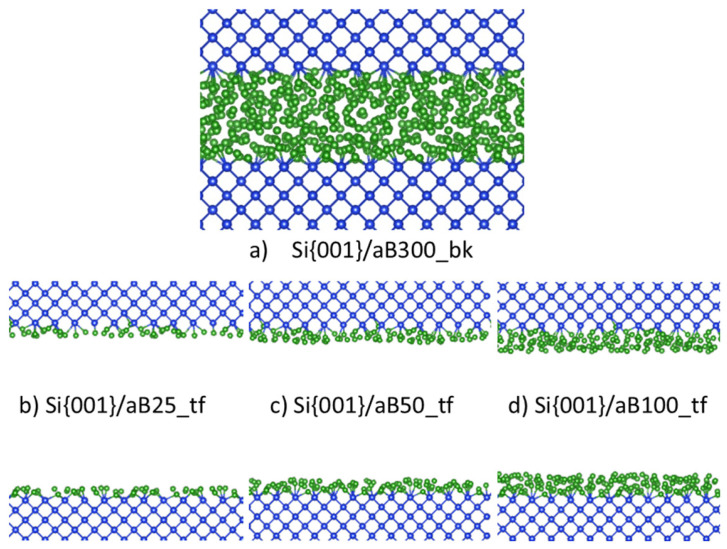
Schematic structures of (**a**) the equilibrated bulk a-B/Si{001} interface system; interface with (**b**) one a-B layer, (**c**) two a-B layers, and (**d**) four a-B layers. The green spheres represent B and the blue spheres Si respectively. The Si-Si and Si-B bonds are included.

To independently obtain an a-B system, we simulated the heating of a pure a-B system at 4000 K, which is above the melting point of B (2348 K). This was left to run for 2000 steps at 1.5 femtoseconds (fs) per step or 3.0 picoseconds (ps) in total, before cooling the system to 1000 K in 4.5 ps. A similar method was used in our prior work to create bulk a-B samples [12]. This slab of a-B was then placed on top of the Si crystal in a supercell. The length of the *c*-axis for this supercell was determined by the thickness of the Si-slab and the a-B layer. Thus, a tetragonal supercell with *a* = 16.40 Å, *c* = 19.19 Å was built. This cell contained eight atomic layers of Si (144 Si atoms) and an a-B layer of 300 B atoms. The built bulk a-B/Si{001} interface system was modeled via an AIMD simulation at 1000 K for 2000 steps, with the substrate Si atoms being pinned. A cross-section of the equilibrated a-B/Si{001} interface system is shown in Figure 1a.

To create atomic-thin a-B layers, the excess a-B atoms were removed in the bulk a-B/Si{001} interface system. Three atomic-thin a-B/Si{001} interface systems were built (see Figure 1), which we indicate as B_X_/Si_144_ (X = 25, 50 or 100), with a-B thickness of about 2 Å per layer. The obtained systems retain the tetragonal cell with *a* = 16.40 Å, *c* = 34.18 Å. With these changes we obtained four a-B/Si{001} interfaces with thin a-B layers with one, two and four a-B layers on the Si substrates (Figure 1b–d, respectively). The vacuum length between the B layers for each system is longer than 15.2 Å. This separation helps to avoid unphysical interactions between the two interfaces in these systems.

### 2.2. Ab Initio Techniques

In this study, we employed the first-principles software VASP (Vienna Ab initio Simulation Package) [22,23] to simulate the a-B/Si interfaces. This software was operating on a Tier-3 HPC (High Performance Computing) cluster. The VASP framework utilizes a pseudopotential plane-wave approach within the density functional theory [24] with the projector augmented wave scheme [25]. The generalized gradient approximation with the scalar relativistic approximation by Perdew, Burke and Ernzerhof (GGA-PBE) was used for the exchange and correlation terms [26].

We used a cut-off energy of 400.0 eV for the wave functions and 550.0 eV for the augmentation functions, respectively, for the duration of the structure relaxation process and electronic structure calculations. These cut-off energies are higher than the corresponding default values of the atoms (*E*_MAX_/*E*_AUG_ = 245.3 eV/322.1 eV for Si and 318.6 eV/535.5 eV for B). Dense *k*-meshes were employed, e.g., 8 × 8 × 1 (32 to 34 k-points), generated using the Monkhorst–Pack scheme for the Brillouin zones of the interface systems [27]. In particular, we chose to focus on analysis of the DOS curves of interfacial atoms.

**Figure 2:**
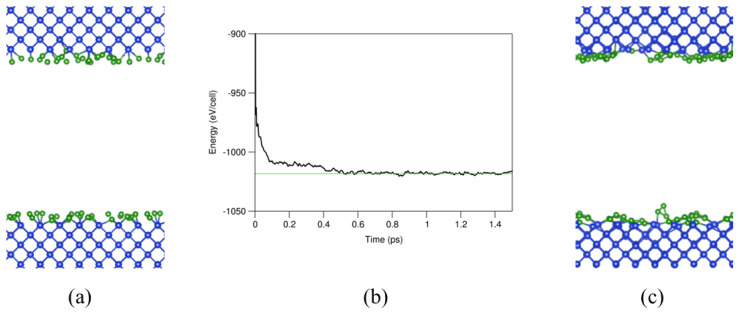
A cross-sectional representation of the (**a**) input and (**c**) equilibrated system. (**b**) The dependence of the total valence-electrons energy of the system on the ab initio molecular dynamics simulation time. The free energy of the simulation nears the convergence value around approximately 0.5 ps. The meanings of the spheres in (**a**,**c**) are the same as those in Figure 1.

For the AIMD simulations, a cut-off energy of 320 eV and the Г (0, 0, 0) point in the Brillouin zone were used, since there is a lack of translational symmetry in the systems with a-B layers. The interface systems were simulated at 1000 K for two to five ps. The ab initio molecular dynamics simulations showed that the interface systems reached equilibrium at about 0.5 ps, as shown in Figure 2b for the one a-B layer system, as an example. The equilibrated systems were then used for further first-principles band structure calculations and local structural analysis.

## 3. Results

### 3.1. Elemental Solids α-B, Si and Amorphous B

**Figure 3:**
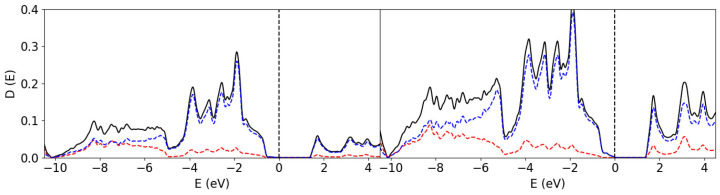
The pDOS of the two species in α-B_12_. The Fermi level is indicated at 0 eV by the perpendicular dotted lines. The red and blue lines represent the s- and p-state contribution to the DOS. The black lines show the total DOS of the system.

In order to make a direct comparison between the properties of B in its crystalline and amorphous forms, we first calculated the electronic properties of the elemental solids α-phase (α-B) and amorphous boron form in an approach that was similarly used in our prior work [12]. The structure of α-B is well-studied and is characterized as a rhombohedral lattice with space group R-3m (Number 166), which contains two groups of B atoms with 12 B atoms in the unit cell (α-B_12_) [28]. The boron atoms in α-B_12_ have six to seven neighbors, with interatomic distances ranging from 1.71 to 2.02 Å. The partial Density of States (pDOS) of the two different species in α-B_12_ are shown in Figure 3.

The DOS curve of α-B_12_ consists of two parts, a valence band from −10.1 eV to the Fermi level, which we set to 0 eV, and a conduction band from +1.7 eV and above. The 2s states dominate the lower part of the valence band from −10.1 eV to −8.0 eV, while the upper part of the valence band and the conduction band are dominated by the 2p states. A bandgap is found for the α-phase, as shown in Figure 3, to be approximately 1.2 eV, which is smaller than the experimental value (1.7 eV) [29]. The underestimation of bandgaps for semiconducting materials is a known factor for density-functional theory methods [30]. Analysis shows that the occupied states near the Fermi level are predominantly B 2p orbitals, with moderate contribution from the s-orbitals.

**Figure 4:**
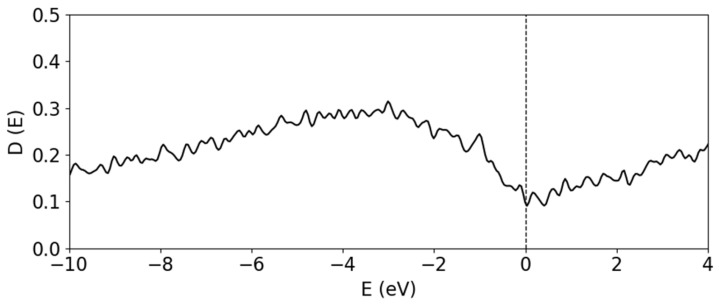
The total DOS of the amorphous bulk B_300_ system.

The a-B system has, by definition, no clear point and translational symmetry. Qualitatively, local structures are found within the system in the form of distorted B_12_ clusters. For a-B there is no clear bandgap, as evident from Figure 4, despite the presence of distorted B_12_ clusters in the sample. Furthermore, the total DOS (tDOS) of amorphous B in Figure 4 shows that the Fermi level falls in a valley with localized defect states. By breaking the symmetry and effectively introducing numerous defects, the bandgap which was opened in the α-B_12_ system is filled with defect states. This leads to the ‘bad’ metal nature of bulk a-B, as found in previous calculations [12].

Finally, to complete the comparison, we simulated crystalline Si. The structure of Si is very well-studied and consists of a face-centered cubic (fcc) lattice (space group Fd-3m, nr. 227) with Si atoms at the (0, 0, 0) and (¼, ¼, ¼) Wyckoff sites. The structure optimization produced a lattice parameter of 5.468 Å for Si. This agrees well with the experimental value (5.4298 Å at 0 K) [20]. The calculated bandgap is 0.7 eV, which is lower than the experimental value (1.17 eV) [31]. The underestimation of bandgaps for semiconductors, including Si, has been widely reported for the DFT-GGA approaches [12,26,30].

### 3.2. Local Structures at the a-B/Si{001} Interfaces

**Figure 5:**
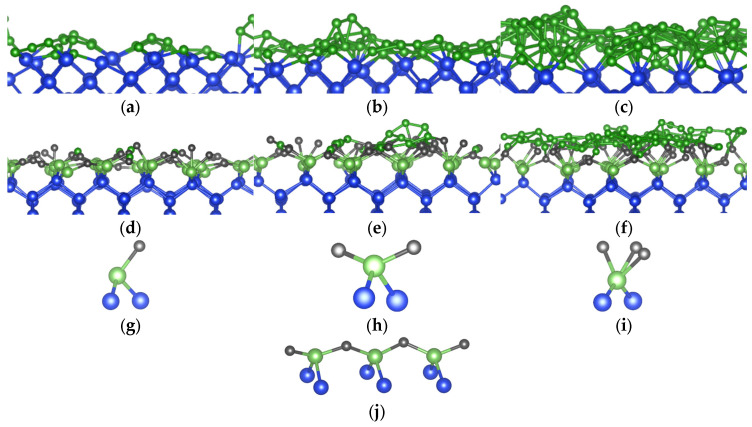
A cross-sectional view from the (100)-plane of the (**a**) B_25_/Si, (**b**) B_50_/Si and (**c**) B_100_/Si systems. (**d**–**f**) The cross-section from the (110)-direction showing the top three layers of c-Si and the Si-B interaction at the interface following relaxation calculations of the atomic positions after removing the excess atoms. Furthermore, a typical representation of Si atom coordination with (**g**) one B atom, (**h**) two B atoms, (**i**) three B atoms and (**j**) a typical (-B-Si-B-Si-) chain. The blue and green spheres represent Si and B, respectively. The gray and light-green spheres represent B and Si atoms bonded to each other at the Si-B interaction layer. The Si-Si, B-B and Si-B bonds with a length of less than 2.6, 1.8 and 2.2 Å respectively are drawn.

Using the parameters and input systems as described in Section 2 we performed ab initio molecular dynamics simulations for the c-Si/a-B interface systems of different a-B thicknesses. Cross-section views of the thermally equilibrated systems are shown in Figure 5a–f in the Si{001} and Si{110} directions. Furthermore, the various local a-B/Si configurations present in these systems are drawn in Figure 5g–j.

At the bulk a-B/Si{001} interfaces, the Si atoms generally maintain the crystalline structure at the interface, while no long-distance ordering is found for the B atoms that are away from the interface (Figure 1a). A slight distortion of the Si crystalline lattice takes place at the surface. This is likely due to a combination of three factors: edge effects from the discontinuation of the lattice, the presence of the B atoms causing a reconstruction of the Si surface, and kinetic effects [10,11,32,33]. The B atoms adjacent to the Si substrates appear to conform to the Si{001} crystal by occupying the vacant Si sites. The thickness of the B layer on top grows with the increasing quantity of B atoms, which is similar to gaseous atoms condensing on a solid surface.

Figure 5a and Figure 5d show the structure and coordination of the interfacial atoms for the B_25_/Si system, respectively. The maximum length of the drawn a-B/Si bonds was chosen to be 2.2 Å based on the covalent radii of the Si and B atoms (1.11 Å and 0.82 Å respectively [34]), with an additional 10% margin to consider the contribution to the chemical bonding according to the valence bond theory [34]. Most of the B atoms are positioned near the original crystalline Si positions. The interfacial Si atoms primarily have two B neighbors and form (-B-Si-B-Si-) chains (Figure 5j). Thus, most B atoms have just two Si neighbors. This is similar to the previously modeled reconstructed Si{001} surfaces with BH_n_ radicals [10,11]. Given the thinness of the B layer in this system, bulk B structures were not expected to form and were not found. Furthermore, Figure 5a also shows that the AIMD simulations resulted in some B atoms moving away from the Si surface, forming a B islands-like structure. This may help understand the island growth of a-B at the Si substrates.

Increasing the thickness of a-B led to more neighbors for the interfacial Si and the B atoms adjacent to the Si substrates (Figure 5b,c,e,f). Analysis revealed that the Si surfaces become less distorted with increasing a-B thickness, which also changes their chemical bonding and related electronic properties. The well-known formation of the B_12_ icosahedral clusters was not observed in the simulated B_25_/Si and B_50_/Si systems. However, smaller B-clusters were found for the B_100_/Si system with four a-B layers, indicating the preference for B-clustering in systems with thicker a-B layers. This is likely due to the Si-B interactions dictating the local structures of the B layer. In other words, the direct interaction between Si and B atoms at the interface leads to different configurations in comparison to those dictated by the B-B bonds. This later induced formation of B_12_ clusters in a-B bulks, as found in the literature [12,35].

**Figure 6:**
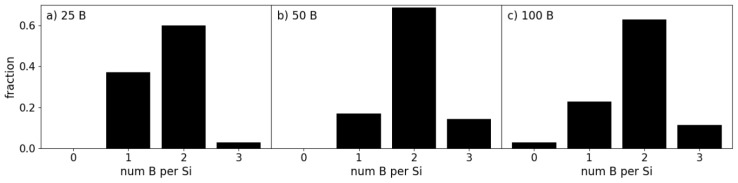
Distribution of the numbers of B atoms coordinating with a Si atom for the (**a**) B_25_,/Si, (**b**) B_50_/Si and (**c**) B_100_/Si interfaces.

The preference for the Si-B interactions is additionally reflected in the coordination of the interfacial B atoms. We define the coverage of the Si layer as the percentage of surface Si atoms interfacing with the number of B atoms. The coverage of the interfacial Si atoms by B atoms for each of the B_X_/Si_144_ (x = 25, 50, 100) systems is shown in Figure 6. Here we find that the coverage is maximal even for the B_25_/Si system. Every surface Si atom has one or more B neighbors in the case of the B_25_/Si and B_50_/Si systems. Meanwhile, there is one Si atom in the B_100_/Si_144_ system with no Si-B bonds.

The distributions of B coordination numbers to Si in Figure 6 correspond to the local structures shown in Figure 5g–i. The Si atoms were found predominantly coordinated to 2 B atoms (1Si:2B) (Figure 5h). These interface Si and atoms formed a zigzag pattern (Figure 5j) on all three interfaces. The Si-B interactions lead to an extension of the Si crystalline structure into the first B layer. This has a profound effect on the electronic properties of the heterojunction, as the electronic properties of the interfacial B and Si atoms share strong similarities. However, note that as the amount of B atoms increases, the amount of Si atoms remains the same. The ratio between the amount of 1Si:1B and 1Si:2B clusters changes only slightly between the increase in B atoms among the B_X_/Si_144_ (x = 25, 50 and 100) systems. The largest change was found between the B_25_/Si_144_ and B_50_/Si_144_ interfaces, as the distribution of coordination shifts even more towards the 1Si:2B configuration (Figure 6b) due to the second a-B layer. In particular, the interface atoms are shielded from the vacuum with the addition of the second a-B layer. This increase corresponds to more B atoms being included in the a-B bulk, which logically means that the electronic properties of the interface are increasingly determined by a-B.

In brief, the B atoms interacting with Si resulted in a preference for the original high-symmetry positions, which relates to the crystal field of the surficial Si atoms, as shown in Figure 5d–f. These B atoms tended towards settling in the planes between the interfacial Si atoms. Additionally, such occupation of these positions was made possible due to the small atomic size of B atoms relative to that of Si. The repulsive charge interaction between the B atoms was partially negated by the attractive force of the nearby Si atoms in this configuration.

### 3.3. Electronic States at the a-B/Si Interfaces

**Figure 7:**
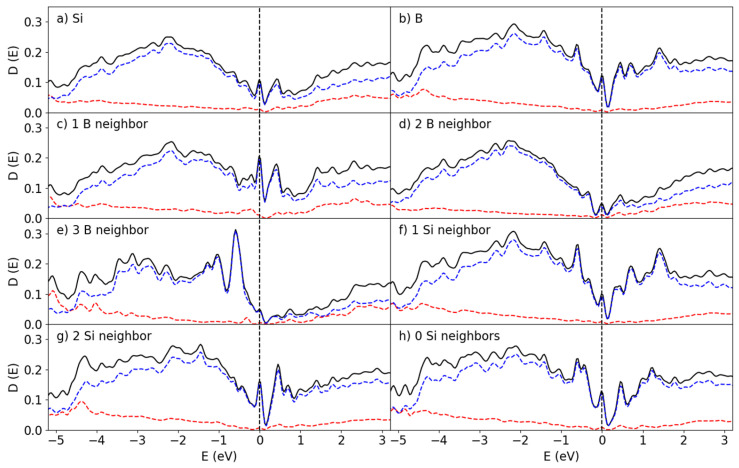
Partial DOS at the B_25_/Si_144_ interface for the (**a**) Si and (**b**) B atoms, the surface Si atoms coordinated with (**c**) 1 B (**d**) 2 B (**e**) 3 B atoms, the interface B atoms interacting with (**f**) 1 Si and (**g**) 2 Si atoms, and (**h**) B atoms without any Si-B interaction. The red and blue lines correspond to the s and p states, respectively. The dotted perpendicular lines represent the Fermi level (at 0 eV).

The interactions between the Si and B atoms at the interface are expected to play a dominant role in determining the electronic properties at this interface. A graph of the summed partial density of states (pDOS) over the atoms at the entire surface and for the individual species found near the B_25_/Si_144_ interface is shown in Figure 7. The localized states at the interface here were found within the bandgap of the Si and B structures. In effect, these states closed the bandgap, which resulted in the formation of a pseudo-gap. The pDOS curves given in Figure 7a,b were averaged out over all the atoms in the entire system. However, the local interactions play a large role in the electronic properties.

Analysis of the eigen-characters near the Fermi level showed that the Si 3s 3p and the B 2s 2p states interact, which in turn is the cause of the bonding between Si and B at the interface. This is reflected in the local structures found in Section 3.2, as the Si crystalline structure partially extends past the interface into the first B layer. The orientational preference of these orbitals is partially responsible for the distribution of coordination numbers in Figure 6, as the Si atoms require two additional bonds at the interface.

The pDOS curves corresponding to the Si atoms in the Si-B configurations found in Figure 5g–i are shown in Figure 7c–e. There is a remarkable difference in DOS depending on the coordination of the Si by B. For the 1Si:2B cluster (Figure 5h), the bandgap in Figure 7d is pronounced though localized states that have been introduced by the Si-B bonding. The 1Si:1B formation (Figure 5g and Figure 7c) has a larger number of states in general near the bandgap compared to 1Si:2B and 1Si:3B (Figure 5i and Figure 7e). The general outlines of each species of Si differ significantly, though all three clusters have an important element in common: A sharp localized peak right at the Fermi level. The 1Si:2B configuration is the most common one for the B_25_/Si_144_ system according to Figure 6, and therefore the pDOS in Figure 7d should primarily determine the average pDOS in Figure 7a, with a major contribution from the 1Si:1B pDOS (Figure 7c). The contribution from the 1Si:3B clusters in this system to the overall properties is very weak due to how few occurrences there are.

The pDOS for the B atoms given in Figure 7f–h shows that the bandgap is closed by localized states and that the Fermi level lands on top of a sharp peak for the B atoms as well. The valley is more prominent and shows that the bandgap is still maintained. A small majority of the B atoms end up in the 1B:2Si ratio, though this distribution is less skewed when compared to 2B:1Si for the Si distributions.

**Figure 8:**
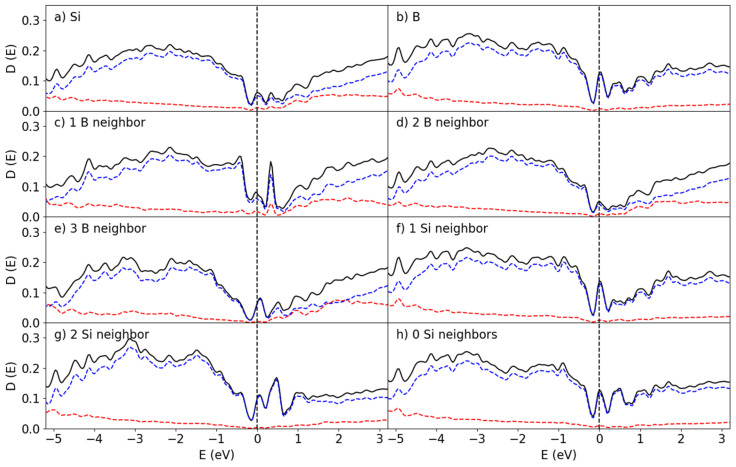
Partial DOS at the B_50_/Si_144_ interface for the (**a**) Si and (**b**) B atoms, the surface Si atoms coordinated with (**c**) 1 B (**d**) 2 B (**e**) 3 B atoms and the interface B atoms interacting with (**f**) 1, (**g**) 2 and (**h**) no Si atoms. The red and blue curves correspond to the s and p states, respectively. The dotted perpendicular lines represent the Fermi level (at 0 eV).

The DOS curves for the B_50_/Si_144_ interface are shown in Figure 8. Clearly they resemble the corresponding ones for the B_25_/Si_144_ system. In both systems the bandgaps disappear for the interfacial Si atoms and sharp peaks arise in place instead from the localized states created by the Si-B interaction. However, unlike the B_25_/Si_144_ system, the Fermi level of the B_50_/Si_144_ system does not lie directly on top of a peak for the 1Si:1B and 1Si:3B configurations, and the height of the peaks are much smaller.

As the symmetry was broken in these systems, the splitting of the degenerate states in the B_25_/Si_144_ and B_50_/Si_144_ systems is unlikely to be caused by structural distortion [36]. This is more likely to be related to the low coordination number of B atoms in the (-Si-B-Si-) chains. The peak at the Fermi level for the interfacial Si atoms comes from the B atoms with low numbers of chemical bonds compared with those of bulk a-B (See Section 3.1).

**Figure 9:**
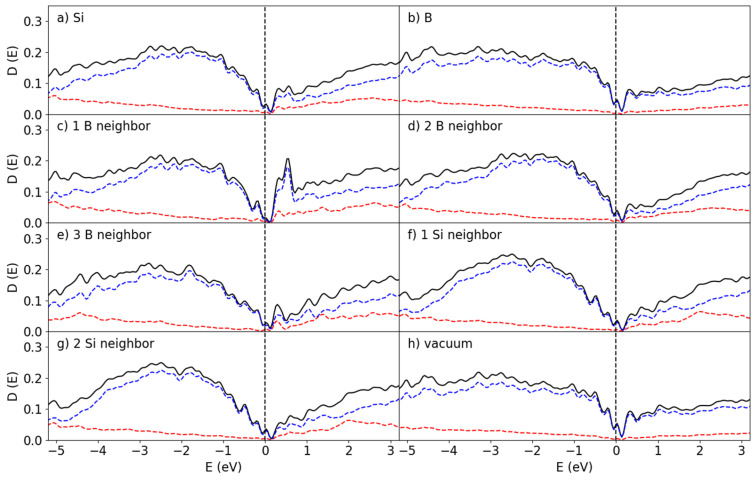
Partial DOS at the B_100_/Si_144_ interface for the (**a**) Si and (**b**) B atoms; the surface Si atoms coordinated with (**c**) 1 B, (**d**) 2 B, and (**e**) 3 B atoms and the interface B atoms interacting with (**f**) 1 Si and (**g**) 2 Si atoms, in addition to (**h**) the pDOS for the B atoms away from the interface. The red and blue curves correspond to the s and p states, respectively. The dotted perpendicular lines represent the Fermi level (at 0 eV).

Finally, the amount of B atoms was doubled again to create the B_100_/Si_144_, for which the DOS is shown in Figure 9. This doubling certainly did not double the amount of surface B with Si-B interactions but manifested the formation of extra layers on top of the B_50_/Si_144_ structure instead. This in turn lead to an increased contribution to the DOS by the non-interface B, which partially corresponds to the B atoms interfacing with the vacuum, as shown in Figure 9h. Aside from this effect, the B_100_/Si_144_ system differs from the previous two with the Fermi level not landing directly on top of a peak, in addition to the associated peak being much smaller.

The bandgap is much more recognizable for this structure, though its width still remains significantly smaller than that found for α-B_12_ and does not resemble the distribution found for B_300_. Instead, the electronic structure of this interface is closer to that found in a prior work for bulk a-B on Si{001} [12]. This is an indication that four layers of a-B already start to exhibit the bulk properties which, realistically, dominate experimental results on devices made with thicker a-B layers.

The surficial B atoms exposed to a vacuum appear to exhibit semiconducting features. Experimental observation of the nature of a-B shows semiconductor properties for surficial a-B, though the bulk should be considered a ‘bad metal’. This is due to the nature of photoemission spectroscopy, as it is a surface-sensitive technique [37,38]. The mean free path of electrons escaping from the solid is about 10 Å for photoelectrons with an energy from 20 eV to 1500 eV [37,38]. Therefore, the bulk properties are hardly detected, and these measuring techniques primarily find the edge effects instead.

## 4. Discussion

The present ab initio molecular dynamics simulations revealed that the a-B atoms adjacent to the Si{001} substrates occupy the high-symmetry sites of the missing Si atoms, extending the crystal structure of the Si substrates. However, the smaller B radius causes the interfacial Si and B atoms to form unique (-Si-B-Si-B) chains and the a-B atoms thus predominantly have two Si neighbors for the systems with one or two a-B layers. Electron band structure calculations showed a high density of states—manifesting as sharp peaks—at the Fermi level for the interfacial Si and B atoms, indicating instability according to Stoner’s criteria [39]. Consequently, the a-B atoms tend to form islands.

Our simulated process is different from chemical vapor deposition, which takes place in N_2_-filled reaction chambers while diborane, B_2_H_6_, is introduced with an inert carrier gas. Prior theoretical work has shown that one B_2_H_6_ molecule decomposes into two BH_3_ radicals and interacts with the hydrogen-passivated Si surface, forming Si-H-BH_3_ clusters that further decompose into BH_n_ (n = 0 to 2) and H_2_ until only Si-B is left [10,11]. We posit that the following interactions prevent doping and create a sharp interface:

1. The larger BH_n_ (n = 1 to 3) radicals require high kinetic energies to penetrate into the bulk of Si.
2. The reaction between BH_n_ and the H atoms on the surface of Si limits the kinetic energy of the radical by acting as a soft landing regardless of the deposition temperature.
3. The bond between Si and B atoms is very strong, preventing further movement of the B atoms after the deposition process.
4. The kinetic energies of the BH_n_ radicals are limited at low temperatures and are not enough to overcome the relatively high diffusion barriers.

It is also noted that the ab initio method used here has limitations with respect to the long-ranged disordering of the a-B films due to the size of the supercell and number of atoms. However, it has provided essential information about the interfacial interaction, chemical bonding and local electronic properties. The resulting B layer mostly stayed on top of the Si surface, similarly to the results found using HR-TEM imaging from samples prepared at low temperatures [8]. The interface interactions are then primarily responsible for the electronic properties of this system, as previously predicted [12]. Therefore, we may be able to exclude the possibility of δ-doping from the B layer creating a junction. However, we are presently unable to conclusively explain the exact mechanism that forms this heterojunction. We further note that in practice, structural defects and other foreign impurities at the Si surfaces can have effects on the diffusion rate of B into Si substrates, though this is outside the scope of this work.

Increasing the thickness of a-B enhances the coordination number for the interfacial a-B atoms, and consequently their electronic properties change to those of bulk a-B/Si interfaces. Broadly speaking, the thin a-B layer systems investigated here can be regarded as intermediate stages of depositing a-B on Si surfaces during the PureB processes [3,4,5,6,8,10,11]. However, realistically it has been found to be very difficult to create such thin layers without severe repercussions with respect to the formation of pinholes [5], and, in practice, it is preferable to grow the B layer to at least a few nm (e.g., 4 nm in [5]) to improve the stability of the device.

Additionally, we find that a certain number of a-B layers is necessary to form the interface between Si and B. The B_50_/Si system shows that the influence of Si extends 2–3 layers into the boron bulk. A thin layer of a-B atomic-layers (~1 nm) has been shown to be enough for the system to exhibit the properties of bulk a-B on Si in principle [5]; we assume, that this we require a minimum of 3–4 layers on top of the interfacing layers. Furthermore, we have to consider that the B layer has two points of contact in this device composition, as a metal layer is used to close the circuit, and based on the Si-B interaction we assume 2–3 layers of interaction between the B and metal atoms. This places a theoretical lower limit on the thickness of the deposited B on the order of 7–10 layers, or approximately 1–2 nm. This is significantly less than the 4 nm found experimentally [5], though the focus of these efforts is on preventing pinholes.

The study on the influence of the thickness of deposited thin films of selected materials may help further understand heterojunctions with different substrates for various applications [5,7,17,18]. Furthermore, the created AIMD methods for handling different thicknesses of thin films on substrates in order to manipulate the properties of the heterojunctions can be applied to find desirable properties for a broad array of applications, e.g., new solar cells [40,41,42].

## 5. Conclusions

Ab initio molecular dynamics simulations and first-principles band structure calculations were performed for a-B/Si{001} interfaces with atomic-thin a-B layers. Though the present ab initio method is limited to the size of the supercell, number of atoms and description of long-ranged disordering of the a-B films, it has provided essential information on interfacial interaction, chemical bonding and local electronic properties. The study revealed that the a-B atoms occupy the original high-symmetry sites, extending the crystal structure of Si at the interfaces. Analysis revealed that the a-B/Si{001} interfaces exhibit a rich variety of atomic coordination for the interfacial Si and B atoms. The single a-B layer a-B/Si{001} interface largely contains (-Si-B-Si-B) chains, and most B atoms have only two Si neighbors. Consequently, electronically, the B atoms have high electron density at the Fermi level, indicating an instability for these B atoms, and they tend to form B islands on the Si substrates. As we increase the thickness of the a-B thin-film, the B atoms adjacent to the Si substrate have more B neighbors and thus become more like those found at the bulk a-B/Si interfaces; thus, they are more electronically stable. Moreover, electronic structure calculations revealed that the interfacial Si and B atoms at the thick a-B/Si interfaces exhibit intrinsic defect states, and the heterojunctions belong to a metal/semiconductor family with a ‘bad’ a-B metal. Finally, this study showed that intrinsically, approximately 1 nm thickness of a-B is the minimum to produce a-B/Si{001} heterojunctions.

## Data Availability

The raw data supporting the conclusions of this article will be made available by the authors on request.
